# Pyrolysis of human feces: Gas yield analysis and kinetic modeling

**DOI:** 10.1016/j.wasman.2018.07.020

**Published:** 2018-09

**Authors:** Tesfayohanes W. Yacob, Richard (Chip) Fisher, Karl G. Linden, Alan W. Weimer

**Affiliations:** a Civil, Environmental, and Architectural Engineering Department, University of Colorado, 428 UCB, ECOT 441, Boulder, CO 80309, USA; b Department of Chemical and Biological Engineering, University of Colorado, 596 UCB, Boulder, CO 80309, USA; c Present address: Department of Engineering, Messiah College, One College Avenue, Suite 3034, Mechanicsburg, PA 17055, USA

**Keywords:** Fecal sludge, Latrine waste, DAEM, Pyrolysis gas, Biomass pyrolysis

## Abstract

Pyrolysis of human feces renders the waste free of pathogens and is a potential method of treating fecal sludge waste collected from non-sewered systems. Slow pyrolysis experiments were conducted on human feces and the char yield and gas evolution quantified at 1–10 °C/min heating rates. Char yield ranged from 35.1 to 35.8% (dry mass basis), while the gas yield ranged from 17.2 to 29.6% (dry mass basis). The pyrolysis gases detected were CO, CO_2_, CH_4_, C_2_H_6_, and H_2_. These non-condensable gases contained a higher heating value (HHV) ranging from 7.2 to 22.8 MJ/Nm^3^. Kinetic analysis was done by a pyrolysis reaction model free method (Isoconversional) as well as a DAEM (Distributed Activated Energy Model) method that assumes many irreversible first order reactions. Both yielded very close values for activation energy ranging from 141 kJ/mol to 409 kJ/mol, with half of the biomass conversion happening at 241.5 ± 2.9 kJ/mol. The findings of the research provide useful technical information that can guide the design of a pyrolysis system to treat fecal waste. Social acceptance and scale-up issues need to be addressed through further research.

## Highlights

• Quantitative TGA-MS used to study pyrolysis of fresh feces.
• An HHV of 7.2–22.8 MJ/Nm^3^ was estimated for the pyrolysis exhaust.
• A model free isoconversional and DAEM methods were used for kinetic analysis.
• Half of the biomass conversion occurred at 241.5 ± 2.9 kJ/mol activation energy.

## Parameter list

$k\left(T\right)$: reaction the rate constant
α: extent of conversion based on gross volatiles
$f\left(\alpha \right)$: is an expression of a reaction model, as a function of α
E: the activation energy
R: the universal gas constant
$k_{0}$: pre-exponential factor (frequency factor)
β: constant heating rate
T: temperature
g(α): integral form of the kinetic expression $f\left(\alpha \right)$
$\Phi \left(E_{\alpha },T\right)$: shorthand for the Arrhenius-type temperature integral, to simply notation/math
$N_{\exp}$: is the number of experiments performed at different heating rates β
i: denotes the $i^{\mathrm{th}}$ experiment
$t_{\alpha }$: time it takes to achieve a conversion of α for an isothermal kinetics
$k_{0}\left(E\right)$: pre-exponential factor (frequency factor) that varies with activation energy
$f\left(E\right)$: expression for the distribution of activation energy
$\Psi \left(E,T\right)$: Coats-Redfern approximation
$E_{s}$: Value of activation energy that best approximates $\Psi \left(E_{S},T\right)$ as a step function

## 1 Introduction

Currently, 40% of the global population lacks access to sanitation services and facilities in part due to the high costs of construction and materials. The majority of new toilets being installed in developing countries are ventilated improved pit (VIP) latrines that do not use flush water. Flush toilets are unsustainable because of the large energy and water requirements for maintaining the infrastructure (e.g. sewers) and wastewater treatment processes (Norman and Chenoweth, 2009). Although consuming low amount of water for their operation, the level of treatment for pit latrine waste is very low in most low-income countries. This is especially pronounced in densely populated urban areas, where the waste material filling up pit latrines is often dumped into the environment. Development of new and improved treatment processes are thus needed for fecal sludge generated from latrines. Processes that can convert the fecal sludge into valuable products in addition to treatment, could create monetary incentives for communities and entrepreneurs to adopt safe sanitation practices in low-income communities.

Pyrolysis is the thermal degradation of organic materials to a carbon-enriched product namely char. It is well known that biomass pyrolysis also yields non-condensable gases of which some give off energy when combusted, as well as energy-rich solid and liquid products (tars and oils) that can be used directly as fuels (Kaminsky and Kummer, 1989, Ward et al., 2014) or chemically converted to higher-grade fuels. Some of the liquid products are released as pyrolysis gases but condense at room temperature and pressure to be liquids, while non-condensable gases remain gases. Agricultural and carbon sequestration benefits of the solid pyrolysis products, char, have also been demonstrated (Lehmann et al., 2006, Spokas et al., 2012). Biochar has also been demonstrated to potentially have the ability to remove pollutants from water (Kearns et al., 2015, Mohanty et al., 2018, Mohanty et al., 2014).

Slow biomass pyrolysis is largely an endothermic process with an enthalpy of 1.3–1.6 kJ/g although depending on the operating conditions, extent of secondary reactions, and sample treatment, exothermic values can be obtained (Gomez et al., 2009, Yang et al., 2013). The use of renewable energy to drive pyrolysis of biomass could make the process more economical and carbon neutral. Previous investigators have shown that concentrated solar-thermal power (CSP) can be used to drive biomass gasification at high temperatures (Lichty et al., 2010). Researchers at the University of Colorado at Boulder have demonstrated a novel human fecal waste pyrolysis prototype powered by CSP (Hernandez, 2014).

Estimation of the gaseous yields from biomass pyrolysis is most often performed by capture and analysis of the pyrolysis exhaust stream (Menéndez et al., 2004). Pyrolysis studies done using thermogravimetric analysis coupled to mass spectrometry (TGA-MS) systems have mostly been limited to qualitative descriptions of the evolved gases.

A variety of animal manures (such as pig, chicken, horse, and cow), as well as agricultural residues, have been shown to yield useful gaseous and solid products when pyrolyzed (Hussein et al., 2017, Kim and Agblevor, 2007, Kim and Agblevor, 2014, Ngo et al., 2010, Tsai et al., 2015). System-level study of using animal manure and pit latrine sludge as a source of energy demonstrated the potential of thermochemical processes in alleviating waste while providing renewable energy (Bond et al., 2018, Cantrell et al., 2012). Thermogravimetric analysis (TGA) of manures allowed for the determination of kinetic expressions that can predict product yields over a given operating temperature range (Kim and Agblevor, 2007, Ro et al., 2009). Liu et al. have performed pyrolysis experiments on partially decomposed septic tank waste (Liu et al., 2014) and showed the energy benefits of pyrolyzing the waste. Researchers have also demonstrated the production of fuel briquettes from the pyrolysis of untreated fecal sludge (Ward et al., 2014).

Robust and accurate models to describe the kinetics of fecal sludge pyrolysis are necessary to design an optimized waste treatment process based on pyrolysis. Different methods for determining the kinetic parameters of biomass pyrolysis have been described in the literature. Simple first-order kinetic expressions are commonly used for biomass pyrolysis kinetics (Kim and Agblevor, 2007). These expressions can be used to describe a single reacting component or multiple reacting components. Model-free methods for determining the conversion dependent activation energy relations have been described by Vyazovkin and Wight (1999) for pyrolysis of various organic materials. Distributed activation energy models (DAEM) extend simple first-order kinetics from single or multiple reactions to a theoretically infinite number of reactions that occur with different kinetic parameters, namely the activation energy (Braun and Burnham, 1987). The accuracy of a kinetic model is largely dependent upon the validity of the so-called kinetic triplet – the activation energy $\left(E\right)$, pre-exponential or frequency factor $\left(k_{0}\;or\;A\right)$, and the reaction order $\left(n\right)$ – over the extent of conversion.

The purpose of the current study is to investigate the slow pyrolysis of un-treated human feces using Thermogravimetric Analysis, quantify the non-condensable pyrolysis gases released, and model the reaction kinetics.

## 2 Materials and methods

### 2.1 Experimental methods

Samples were prepared from stool collected from a healthy adult male and female over a period of several days. Stool was collected in its entirety (without subsampling) and later dried in an oven at 105 °C, crushed, mixed well, and sieved to ≤422 μm (US #40 mesh size), and then stored in a desiccator until pyrolysis. The experiments were run in a thermobalance (Netzsch STA 449F1 Jupiter) coupled with a quadrupole mass analyzer (Netzsch QMS 403C Aëolos) to detect evolved gasses during the experiments. Approximately 100 mg of the dried and sieved samples were loaded into an alumina TG-DTA crucible for each pyrolysis experiment. Any air in the thermobalance was purged with vacuum and inert gas backfill and then the samples were heated at five different linear temperature ramp programs: 1, 2.5, 5, 7.5, and 10 °C/min. Low heating rates were used in order to represent what could be achieved in low-cost biomass pyrolizers.

Pyrolysis proceeded under an argon flow of 60 SCCM. The inert gas minimizes the possibility of secondary vapor phase reactions of the released pyrolysis gases. Signals for mass loss (TG), temperature, time, and the ion current values for various mass numbers (MS) corresponding to known biomass pyrolysis gases were collected throughout each experiment. The analysis was focused on non-condensable pyrolysis gases and specifically H_2_, CH_4_, CO, CO_2_, and C_2_H_6_. Mass numbers were chosen based on known fragmentation patterns, where m/z=2 (H2), 15(CH_4_), 12(CO), 30(C_2_H_6_), and 44 (CO_2_). The ion current values of the chosen mass numbers were converted to concentrations of H_2_, CH_4_, CO, CO_2_, and C_2_H_6_ using calibration curves generated from standard gas mixes (0.02%, 0.2%, and 1%). The ion current signal for CO was corrected to account for the interference from CH_4_ and CO_2_ using the fragmentation patterns. The transfer line between the TGA and the MS was heated to 230 °C in order to prevent condensation of volatile species evolved during pyrolysis and the MS capillary probe was placed very close to the sample crucible of the TGA in order to avoid detection of secondary reactions. Any buoyancy effects from gas flow through the thermobalance were corrected with blank runs at the normal experimental gas flow and temperature conditions.

Mass loss data were normalized to the initial mass for TG and the derivative TG (DTG) plots and to the final mass (i.e. on the basis of volatiles evolved) for kinetic modeling. DTG, or rate of mass loss, was computed by two-point slope calculation from the mass remaining and time (minutes) data and smoothing using the Sovitzky-Golay filter method. To visualize multiple gas release patterns at a given heating rate, the highest absolute ion current value for each gas was used to normalize the ion current readings for that specific gas over the range of operation temperatures. The total flow of gas through the system was updated based on the concentration of gases detected at each time step and used to calculate the amount of pyrolysis gas released in moles. The moles of gas evolved were converted to mass to calculate the mass yield of the gases from the initial sample mass. Calculation of the actual concentration of the pyrolysis gases, as if the inert gas were not present, was performed by multiplying the measured concentrations by a dilution factor of the inert gas used, calculated as the ratio of the total gas flow to the difference of the total gas flow and the argon flow.

C, H, and N content in the dried and sized feces samples were analyzed by Perkin-Elmer model 2400 elemental analyzer at the Analytical Services Laboratory of North Carolina State University. Analysis for S, Ca, P, K, and Si was conducted using ICP-OES at the LEGS laboratory of the Geological Sciences Department, at the University of Colorado Boulder. Ash content was determined by heating pre-dried and desiccator kept samples in the following program according to NREL/TP-510-42622 (Sluiter et al., 2005): holding it at 105 °C for 12 min, ramp to 250 °C at 10 °C/min, hold at 250 °C for 30 min, ramp to 575 °C at 20 °C/min, and hold at 575 °C for 180 min, and cooling down to 105 °C. Gross calorific value (HHV) was estimated from the elemental analysis result using an empirical model developed by Channiwala and Parikh (2002).

### 2.2 Modelling methods

The goals of kinetic modelling are to determine an expression that accurately predicts the rate of reaction for a process and represents the underlying physics with fidelity. Kinetic expressions for thermal decomposition reactions, such as pyrolysis, are typically reported using parameters that define the rate of reaction’s dependence upon temperature, T, and extent of conversion, α, as shown by

(1) $$\frac{d\alpha }{\mathrm{dt}}=k\left(T\right)f\left(\alpha \right)$$

where $k\left(T\right)$ is the rate constant in min^−1^ and $f\left(\alpha \right)$ is an expression for the reaction model in the process. The rate constant, $k\left(T\right)$, is typically assumed to follow the Arrhenius expression

(2) $$k\left(T\right)=k_{0}\exp\left(\frac{-E}{\mathrm{RT}}\right)$$

in which $k_{0}$ is the frequency factor min^−1^, E is the activation energy in J/mol, and R is the universal gas constant in J/mol K. Thermal analysis techniques do not allow for mass loss measurements of an individual reacting species in biomass so overall extent of conversion is determined on the basis of gross volatiles evolved

(3) $$\alpha =1-\frac{m\left(t\right)-m_{f}}{m_{0}-m_{f}}$$

where $m\left(t\right)$ is the sample mass at time t, $m_{0}$ is the initial sample mass, and $m_{f}$ is the final sample mass such that α varies from zero to one. Furthermore, thermal analysis is usually performed under non-isothermal conditions with a constant heating rate (β)

(4) $$\beta =\frac{\mathrm{dT}}{\mathrm{dt}}=constant$$

Incorporating β into the kinetic expression reconfigures Eq. (1) without any explicit temporal dependence

(5) $$\frac{d\alpha }{\mathrm{dT}}=\frac{k_{0}}{\beta }\exp\left(\frac{-E}{\mathrm{RT}}\right)f\left(\alpha \right)$$

forming the basis for all integral kinetic methods that were used in the present study. Treatment of the reaction model $f\left(\alpha \right)$ depends on the kinetic method being employed and the method’s assumptions about how pyrolysis proceeds.

#### 2.2.1 Isoconversional method

Activation energy is a constant value for an elementary reaction step (Vyazovkin, 1997), but, biomass pyrolysis is quite complex with many elementary reaction steps taking place throughout the process. Isoconversional methods can be used to reveal the nature of the reaction mechanism in a multi-step complex reaction. The isoconversional assumption asserts that the reaction model, $f\left(\alpha \right),$ is not dependent upon temperature, or heating rate, and that the activation energy can vary with extent of conversion. This assumption has some useful consequences, one being that the investigator does not have to explicitly assume a functional form for the reaction model, f(α), in order to findE. The isoconversional method helps avoid the short coming observed when obtaining multiple *E* and *K_o_* values with little regression error for completely different reaction models. In addition, activation energy is an intrinsic parameter, and obtaining it independent of an assumed reaction model and heating rate improves the validity of the result (Botas et al., 2012).

By rearranging and integrating Eq. (5), the integral form of the kinetic expression, g(α), is described by

(6) $$g\left(\alpha \right)=\int \frac{d\alpha }{f\left(\alpha \right)}=\frac{k_{0}}{\beta }\int_{0}^{T}\exp\left(\frac{-E}{\mathrm{RT}}\right)dT$$

where α is the reaction extent of conversion, f(α) is some reaction model function, T is the temperature, $k_{o}$ is the pre-exponential factor (frequency factor), β is the heating rate, and R is the universal gas constant. It is also useful to denote the Arrhenius-type temperature integral on the r.h.s. in Eq. (7) as $\Phi \left(E_{\alpha },T\right)$ for a simplified notation.

(7) $$\Phi \left(E_{\alpha },T\right)=\int_{0}^{T}\exp\left(\frac{-E_{\alpha }}{\mathrm{RT}}\right)dT$$

A direct consequence of the isoconversional assumption is that a ratio of the temperature integral for any two non-isothermal experiments to their respective heating rate, β, will be a constant. Now this effect can be used to find the dependence of activation energy on extent of conversion. For $N_{\exp}$ reaction experiments performed at different heating rates, $\beta_{i},$ the activation energy, $E_{\alpha }$, at a given conversion, α, can be found by minimizing the objective function (Vyazovkin and Wight, 1999)

(8) $$\mathrm{obj}=\sum_{i=1}^{N_{\exp}}\sum_{j\neq i}^{N_{\exp}}\frac{\Phi_{i}\left(E_{\alpha },T_{i}\right)\beta_{j}}{\Phi_{j}\left(E_{a},T_{j}\right)\beta_{i}}$$

$$\mathrm{obj}=\frac{\Phi_{1}\left(E_{\alpha },T_{1}\right)\beta_{2}}{\Phi_{2}\left(E_{\alpha },T_{2}\right)\beta_{1}}+\frac{\Phi_{1}\left(E_{\alpha },T_{1}\right)\beta_{3}}{\Phi_{3}\left(E_{\alpha },T_{3}\right)\beta_{1}}+\cdots +\frac{\Phi_{5}\left(E_{\alpha },T_{5}\right)\beta_{4}}{\Phi_{4}\left(E_{\alpha },T_{N_{4}}\right)\beta_{5}}$$

where $N_{\exp}$ is the number of experiments performed at different heating rates β, the index i denotes the $i^{\mathrm{th}}$ experiment, and the index j ensures that $\Phi_{i}\left(E_{\alpha },T_{i}\right)\neq \Phi_{j}\left(E_{a},T_{j}\right)$.

Since the temperature integral Φ does not have an analytical solution, it is common to approximate the solution. It has been shown by Vyazovkin and Dollimore (1996) that rational functions can accurately approximate the temperature integral to within 0.02% of the numerical solution and that these approximations can be up to 10^4^ times faster to compute than a numerical solution. The 3rd order Senum & Yang Approximation (Senum and Yang, 1977) makes use of the substitution u=E/RT

(9) $$p\left(u\right)=\left(\frac{e^{-u}}{u}\right)\left(\frac{u^{2}+10u+18}{u^{3}+12u^{2}+36u+24}\right)$$

and was used as the approximate solution to the Arrhenius temperature integral in the present study.

Finally, by following the methods described by Vyazovkin and Wight (1999), the isothermal kinetics of the feces pyrolysis can be accurately predicted by the following model-free relation

(10) $$t_{\alpha }=\frac{\int_{0}^{T_{\alpha }}exp\left(-E_{\alpha }/RT\right)dT}{\beta exp(-E_{\alpha }/RT_{0})}$$

This would be extremely useful in the case of a continuous pyrolysis process where near isothermal conditions exist, i.e. very high heating rates, inside a pyrolysis reactor. If necessary, Vyazovkin and Wight (1999) mention methods for determining a reaction model, $f\left(\alpha \right)$, and frequency factor, $k_{0}\left(\alpha \right)$, that best fits non-isothermal data. In this presented work, only the activation energy is presented for the isoconversional method since the method described next (DAEM) was used to obtain the frequency factor ($k_{0}$).

Without knowledge of the reaction model or the frequency factor, Eq. (10) can be used to find the time at which a given conversion will be achieved for a reaction occurring at any particular isothermal process temperature $T_{0}$ within the range of temperatures from the experiments.

#### 2.2.2 Distributed activation energy model

It is widely accepted that the kinetics of pyrolysis of complex materials, such as biomass, are well described by the distributed activation energy model (DAEM), initially proposed by Vand (1943) and modified for biomass pyrolysis by Avni et al. (1985). The DAEM assumes that the pyrolysis process consists of many irreversible first-order decomposition reactions that occur simultaneously, and where each reaction has its own activation energy that follows a continuous distribution. So, for first order kinetics, $f\left(\alpha \right)=1-\alpha$, and Eq. (1) can be integrated to form the general kinetic expression for the DAEM (Miura, 1995).

(11) $$1-\alpha \left(t\right)=\int_{0}^{\infty }exp\left(-k_{0}\left(E\right)\int_{0}^{t}e^{-\frac{E}{\mathrm{RT}}}dt\right)f\left(E\right)dE$$

where E is a given reaction’s activation energy, $k_{0}\left(E\right)$ is a pre-exponential factor (frequency factor) that varies with the value of E, and $f\left(E\right)$ is defined to be a distribution of activation energy, such as a Gaussian distribution, that satisfies

(12) $$\int_{0}^{\infty }f\left(E\right)dE=1$$

For non-isothermal pyrolysis with a constant heating rate, Eq. (4) can be incorporated into Eq. (11) to remove explicit temporal dependence from the expression

(13) $$1-\alpha \left(T\right)=\int_{0}^{\infty }exp\left(-\frac{k_{0}\left(E\right)}{\beta }\int_{0}^{T}e^{-\frac{E}{\mathrm{RT}}}dT\right)f\left(E\right)dE$$

Miura (1995) presented a simple variation of the distributed activation energy model that does not require an assumption as to the functional form of $f\left(E\right)$ or $k_{0}\left(E\right)$ and also does not require non-linear least squares optimization. Using the modified Coats-Redfern approximation to the temperature integral, Eq. (7) can be simplified to

(14) $$\Phi =\int_{0}^{T}\exp\left(-\frac{E}{\mathrm{RT}}\right)dT≅\frac{RT^{2}}{E}\exp\left(-\frac{E}{\mathrm{RT}}\right)$$

and then the integrand in Eq. (13) becomes

(15) $$\Psi \left(E,T\right)≅\exp\left(-\frac{k_{0}RT^{2}}{\beta E}e^{-\frac{E}{\mathrm{RT}}}\right)$$

Then, because the slope of $\Psi \left(E,T\right)$ is quite steep for a given temperature and heating rate, it can be approximated by the step function located at an activation energy,$E_{s}$, that equals 1 when integrated from E_s_ to ∞ thus simplifying Eq. (13) to

(16) $$1-\alpha \left(T\right)≅\int_{E_{s}}^{\infty }f\left(E\right)dE=1-\int_{0}^{E_{s}}f\left(E\right)dE$$

Next, Miura and Maki develop an approximation for the hypothetical $j^{\mathrm{th}}$ reaction of the many reactions occurring under the assumptions of the DAEM

(17) $$\frac{d\alpha_{j}}{\mathrm{dt}}≅k_{0}\exp\left(-\frac{E_{s}}{\mathrm{RT}}\right)\left(1-\alpha_{j}\right)$$

where the actual pyrolysis process consists of j=1,⋯,N reactions all at their own temperature and where$\alpha_{j}$ is the volatiles evolved by the $j^{\mathrm{th}}$ reaction. Now, $k_{0}$ and $E_{s}$ are constants in the $j^{\mathrm{th}}$ reaction, so Eq. (16) can be integrated for a constant heating rate

(18) $$1-\alpha_{j}\left(T\right)=exp\left(\left({-k}_{0}/\beta \right)\int_{0}^{T}e^{-\frac{E_{s}}{\mathrm{RT}}}dT\right)≅\Psi \left(E_{s},T\right)$$

and then Miura and Maki chose $E=E_{s}$ so the r.h.s. of Eq. (18) best satisfies the approximation of $\Psi (E_{s},T)$ by a step function, giving a value: $\Psi \left(E_{s},T\right)=0.58$. The value of 0.58 resulted in a minimum error when approximating the cumulative distribution function for activation energy, by a step-function (Miura, 1995). This eventually allows the estimation of f(E), $k_{0}$, and E from an experimental data of α and T. Now, substituting Eq. (15) into Eq. (18) and re-arranging the terms gives

(19) $$\ln\left(\frac{\beta }{T^{2}}\right)=\ln\left(\frac{k_{0}R}{E}\right)+0.6075-\frac{E}{R}\frac{1}{T}$$

Following the method described by Miura and Maki (1998), the distribution of values for $E,f\left(E\right),$ and $k_{0}\left(E\right)$ can be determined from the slope and intercept between experiments run at different heating rates for a given set of conversion values. For each of the selected conversion values, a plot of $\ln\left(\frac{\beta }{T^{2}}\right)vs.\left(\frac{1}{T}\right)$ was made and the slope used to calculate E and the intercept term used to calculate $k_{0}$. f(E) values were calculated from the derivation of the conversion vs. activation energy relationship.

## 3 Results and discussion

### 3.1 Experimental results

**Fig. 1:**
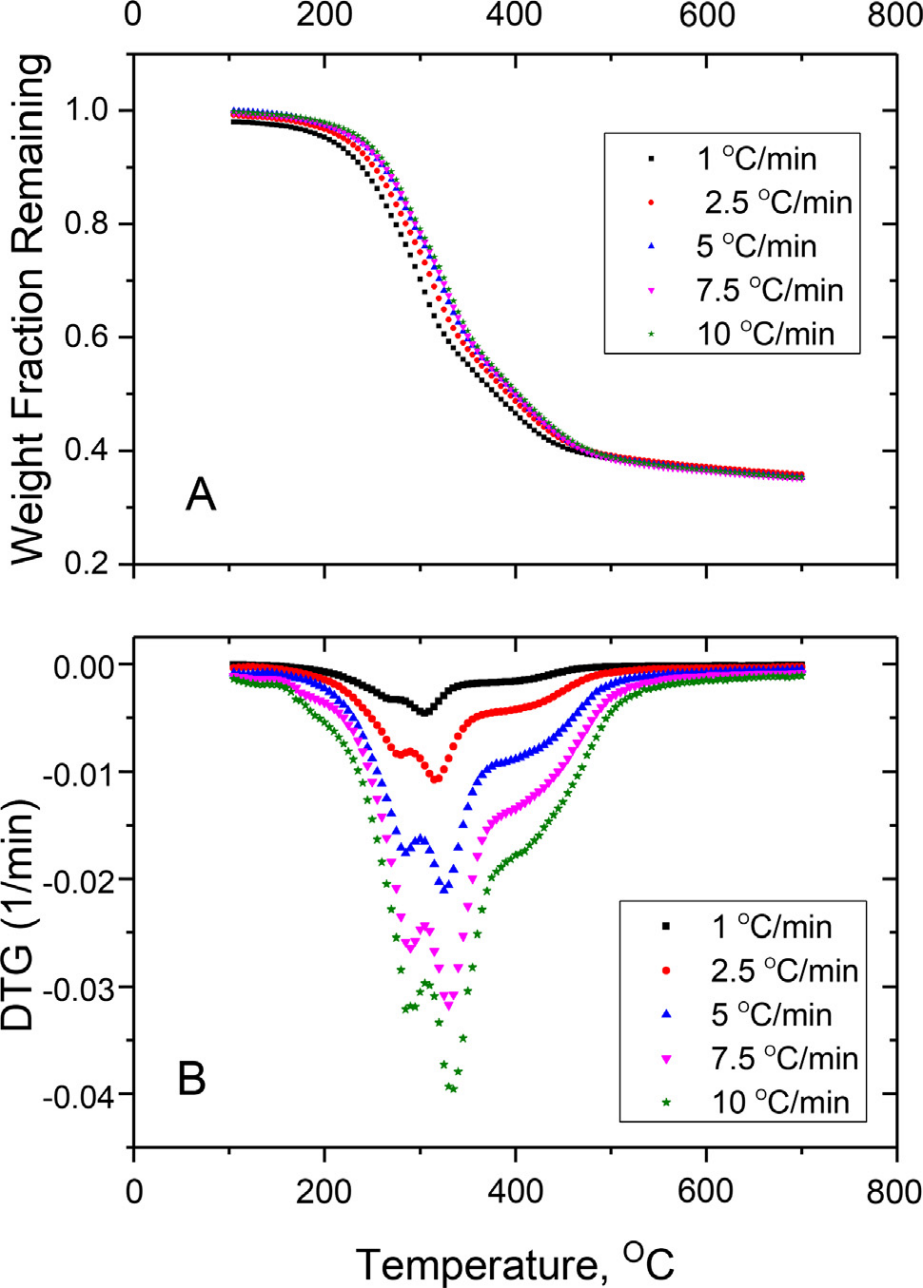
(a) Thermogravimetric curve (TG) plotted as the weight fraction remaining vs. temperature for all four heating rates. (b) The rate of mass change DTG (1/min) plotted as a function of temperature.

The dried and sized samples used for the pyrolysis experiments were analyzed for their elemental and ash content. The results are summarized in Table 1. The conversion of the samples during the pyrolysis process was similar at all heating rates. The mass fraction of the original sample remaining at 700 °C ranged from 0.351 to 0.358. The maximum rate of mass loss (DTG) for the 1 °C/min heating rate occurred at 309 °C with the trough shifting to a higher temperature as the heating rate increased, getting to 332 °C for the 10 °C/min heating rate. Another minor trough was present on the DTG on the range of 271–292 °C in all experiments. The main features of the DTG curves were similar for all heating rates examined (Fig. 1(A)). Other biomass pyrolysis studies with sewage sludge have shown a lowest trough for the DTG between 300 °C and 320 °C, similar to the current study (Conesa et al., 1998). From Fig. 1(B), it can be seen that the lowest trough corresponding to the lowest and highest heating rates were −4.62 * 10^−3^ min^−1^ and −4.03 * 10^−2^ min^−1^, respectively.

**Table 1:** Elemental characteristics of the feces sample used (dry basis).

C (%)	43.47
H (%)	6.42
N (%)	4.57
S (%)	0.65
O (%)	30.05
Ca (%)	3.5
P (%)	2.72
K (%)	0.98
Si (%)	0.68
Ash Content (%)	14.8
HHV, MJ/Kg	19.31

**Fig. 2:**
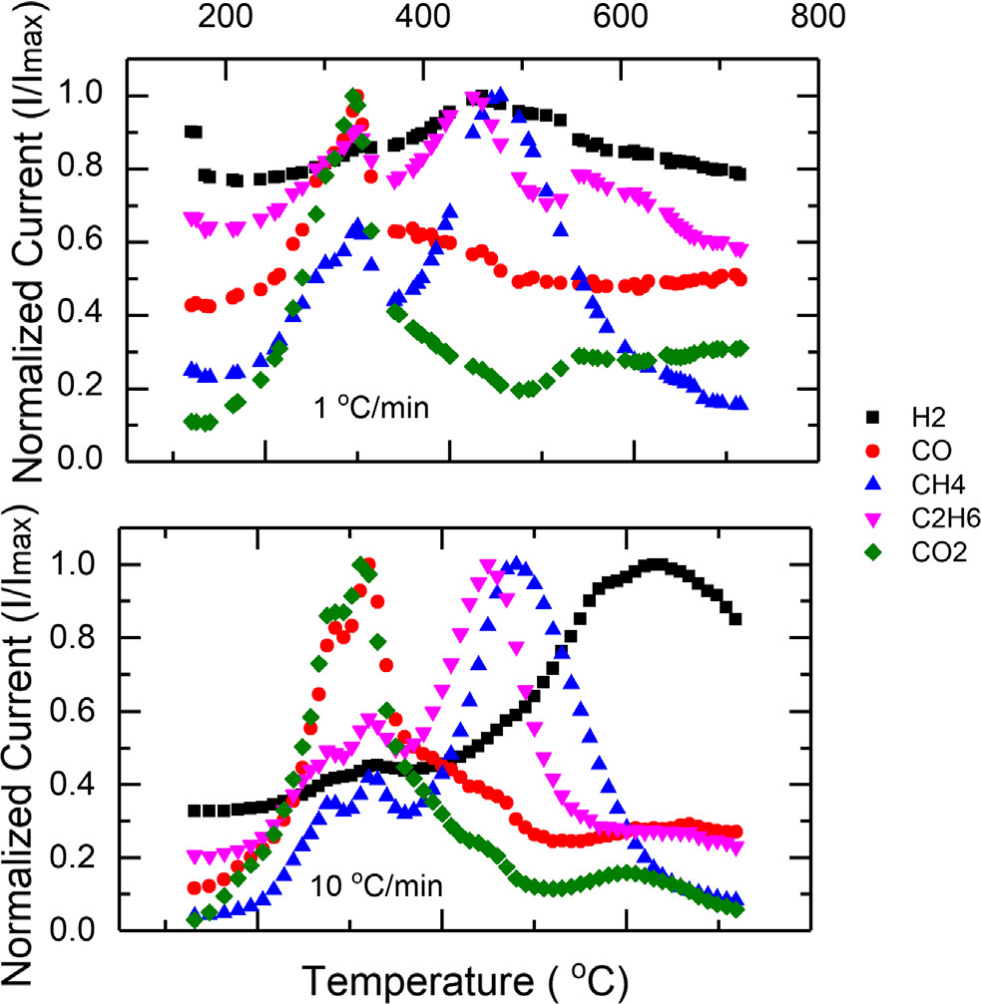
Normalized ion current values of measured pyrolysis gases plotted as a function of temperature for the 1 and 10 °C/min heating rates used.

The first major gas evolution measured was for CO and CO_2_, with the CO having a peak between 299 °C and 320 °C, corresponding to the lowest and highest heating rates, respectively (Fig. 2). A similar range of maximum CO release was shown in a pyrolysis study done on anaerobic sewage sludge with a heating rate of 5 °C/min (Inguanzo et al., 2002). The CO_2_ peak occurred at 299 °C for the lowest heating rate and at 310 °C for the 10 °C/min heating rate. Both CO and CO_2_ featured a smaller peak around 275 °C and a plateau following that until 292 °C corresponding to the smaller DTG trough. The other gas species measured, C_2_H_6_, CH_4_, and H_2_ had local peaks at both of these temperature groups. C_2_H_6_ had the highest peak extending from 424 °C to 450 °C for the various heating rates. This peak temperature range didn’t correspond to any DTG peak. Methane had a major peak within the 444–479 °C range, with the peak time increasing from a low to a high heating rate. Previous studies with sewage sludge had shown a similar peak for C_2_H_6_ but a higher (600 °C) peak for CH_4_ (Inguanzo et al., 2002). It should be noted that the present work does not claim to have detected all non-condensable gases that comprise the pyrolysis product. Based on previous work on sewage sludge pyrolysis, a small portion of the pyrolysis exhaust could be comprised of C_3_ and higher hydrocarbons (Conesa et al., 1998). Unfortunately, fragmentation of higher hydrocarbons in the Mass Spectrometer complicated analysis of these compounds.

**Fig. 3:**
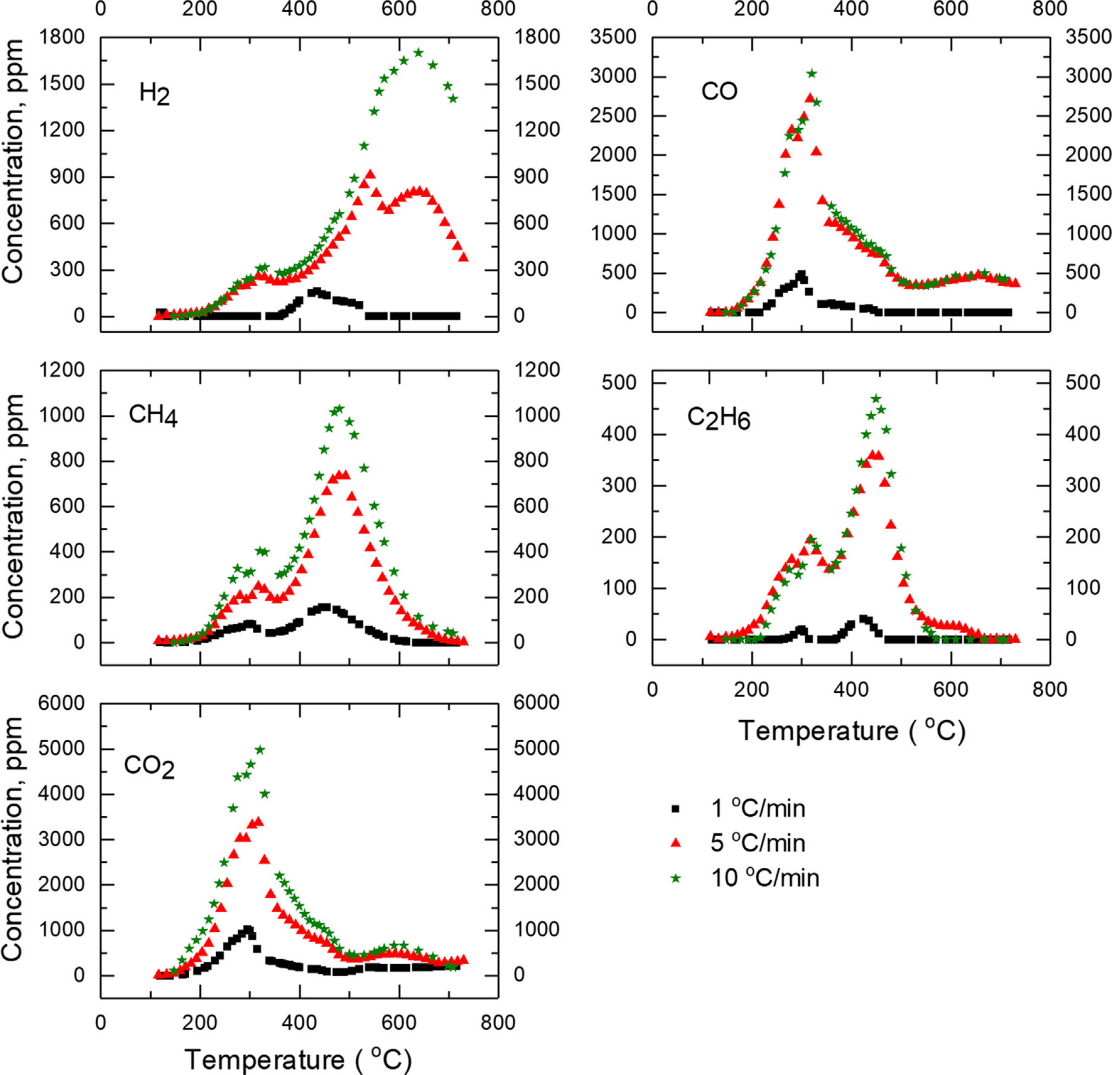
Measured concentrations (ppm) of the pyrolysis gases plotted as a function of temperature for the 1, 5, and 10 °C/min heating rates used.

For most heating rates, the concentration of the gases detected, except hydrogen, resorted to a background level as the temperature approached 700 °C. Similar trends of returning to background levels at higher temperatures were seen in other animal manure pyrolysis studies (Conesa et al., 1998). Hydrogen had its highest peaks at 517 °C, 542 °C and 561 °C for the 2.5, 5, and 7.5 °C/min heating rates. The 5 and 7.5 °C/min heating rates featured a smaller peak around 640 °C. For the highest heating rate, 10 °C/min, the hydrogen major peak occurred around 640 °C (Fig. 3). The hydrogen concentrations didn’t get back to a background value by the time the pyrolysis was completed, in the range of 700–740 °C. The highest concentration of gas detected was CO_2_ with just over 5000 ppm (0.5%), while ethane had the lowest with an order of magnitude less (Fig. 3). The gas concentrations provided in Fig. 3 are only for relative comparison purposes and should not be taken as true concentrations. This is because the pyrolysis exhaust is diluted by the 60 SCCM argon gas flow and is much more than the stated concentrations in non-purged systems.

As described in the methods section, a calculation of non-diluted concentration of the pyrolysis gases was made based on the inert gas flow rate and the non-condensable gas concentrations measured. Table 2 contains the calculated gas concentrations for the 10 °C/min heating rate for selected temperature values and the cumulative mass of gas produced per mg sample at each temperature, and the higher heating values (HHV) of the gas stream. The results show a CO peak of 35 mol% at 330 °C. The peak concentration for CO_2_ is seen to be about 60 mol% at 300 °C. For hydrogen, a continuously rising concentration is seen with 60–70 mol% in the range of 650–700 °C. CH_4_ peaks at 35 mol% and a temperature of 500 °C with most temperatures seeing a concentration below 20 mol%. A sewage sludge pyrolysis study reported lower peak concentrations of 45%, 30%, and 25 mol% for CO_2_, H_2_, and CH_4_, respectively (Inguanzo et al., 2002). The differences could be attributed to the different biomass characteristics and the methods utilized in converting actual measured gas concentrations that are diluted by inert gas to inert gas free concentrations.

**Table 2:** Inert (Argon) gas flow corrected concentrations (mol%) of pyrolysis gases for 10 °C/min heating rate, cumulative gas produced at each of the temperatures, and calculated HHV values are given for selected temperatures.

Temp, °C	CO	CO_2_	C_2_H_6_	CH_4_	H_2_	Cumulative Gas	
		mol%				μmol/mg sample	HHV (MJ/Nm^3^)
300	31.2	59.8	1.8	4.0	3.2	1.56	7.2
350	31.9	52.4	2.9	6.6	6.2	2.69	9.5
450	22.1	28.1	12.8	23.2	13.8	3.83	22.8
600	14.3	22.8	0.0	8.6	54.2	5.18	12.2
700	19.9	10.0	0.0	2.2	67.8	5.99	12.1

HHV was calculated for the pyrolysis gas concentrations corresponding to the 10 °C/min heating rate and are shown in Table 2. HHV represents the maximum energy that can be obtained from a fuel source as it includes the latent heat stored by the vaporized water. Values of HHV for the gases CO, C_2_H_6_, CH_4_, and H_2_ were obtained from a reference handbook (Green and Perry, 1973). The HHV ranged from 7.2 to 22.8 MJ/Nm^3^, with 450 °C corresponding to 22.8 MJ/Nm^3^.

The total amount of gases released per mg of sample for all heating rates is listed in Table 3. In all gases measured except CO, the maximum amount of gas was released at the 2.5 °C/min heating rate. There was low variability on the amount of gases released for the subsequent heating rates (5, 7.5, and 10 °C/min). For CO, the maximum was at the 5 °C/min heating rate. For individual gas species, ethane had the lowest amount released with the next higher release being methane. The most amount of gas released during the pyrolysis of feces at all heating rates was CO_2_. The concentrations of gases showed significant variability over the range of heating rates used (Fig. 3). The peak CH_4_ and H_2_ concentrations were 40 and 90% greater at 10 °C/min compared to the 5 °C/min heating rate. The concentrations at 5 °C/min for all gases were much higher than at 1 °C/min for all gases (232–770%). The increase in concentration is much less at a heating rate of 10 °C/min compared to 7.5 °C/min (1–8%). The exception to this was hydrogen which had an increase of 38%. Concentration has an important implication for safe handling of the pyrolysis gases as it affects the HHV and its combustibility.

**Table 3:** Total amount of non-condensable gases released (µmol/mg sample) for the various heating rates.

	H_2_	CO	CH_4_	C_2_H_6_	CO_2_	Total	Gas Mass Yield (Dry Basis), %
Heating rate °C/min	μmol/mg sample						
1	1.22	0.85	0.66	0.06	3.38	6.18	18.8%
2.5	2.01	1.73	0.98	0.40	4.92	10.03	29.6%
5	1.17	2.27	0.69	0.31	2.77	7.21	20.8%
7.5	1.20	1.59	0.67	0.22	2.66	6.34	18.1%
10	1.25	1.52	0.63	0.20	2.52	6.12	17.2%

Average	1.37	1.59	0.73	0.24	3.25	7.18	20.9%
Standard Dev	0.36	0.51	0.14	0.12	0.99	1.65	5.1%

The cumulative amount of gas released showed that greater than 45% of the total hydrogen evolved was released above 600 °C (Table 4). In comparison, only 10% or less of CO, CO_2_, C_2_H_6_, and CH_4_ were released above 600 °C. Water gas and water shift reactions are responsible for the early release of CO and CO_2_. Nearly half of the total CO and CO_2_ had been released before 350 °C and greater than 75% before 450 °C. For CH_4_, 42% of the total amount was released below 450 °C. Ethane followed a more or less similar trend with CH_4_. An average of 20.2% non-condensable gas mass yield (dry basis) was obtained for all the heating rates (Table 3), while a 44.4% average tar and oil yield was calculated based on the difference between the initial sample and the sum of the char and non-condensable gas yields. This result was in line with other pyrolysis studies done on sewage sludge (Inguanzo et al., 2002, Karayildirim et al., 2006). A pyrolysis study on a partially decomposed waste from a septic tank gave a char yield of 31% by mass (Liu et al., 2014) compared with the current study average of 35.3%.

**Table 4:** The cumulative amount of pyrolysis gas release for the 10 °C/min heating rate.

Temp, °C	CO	CO_2_	C_2_H_6_	CH_4_	H_2_
350	40%	51%	18%	14%	5%
450	76%	83%	67%	42%	15%
600	89%	94%	100%	94%	54%
720	100%	100%	100%	100%	100%

### 3.2 Modelling results

Supplementary data associated with this article can be found, in the online version, at https://doi.org/10.1016/j.wasman.2018.07.020.

**Fig. 4:**
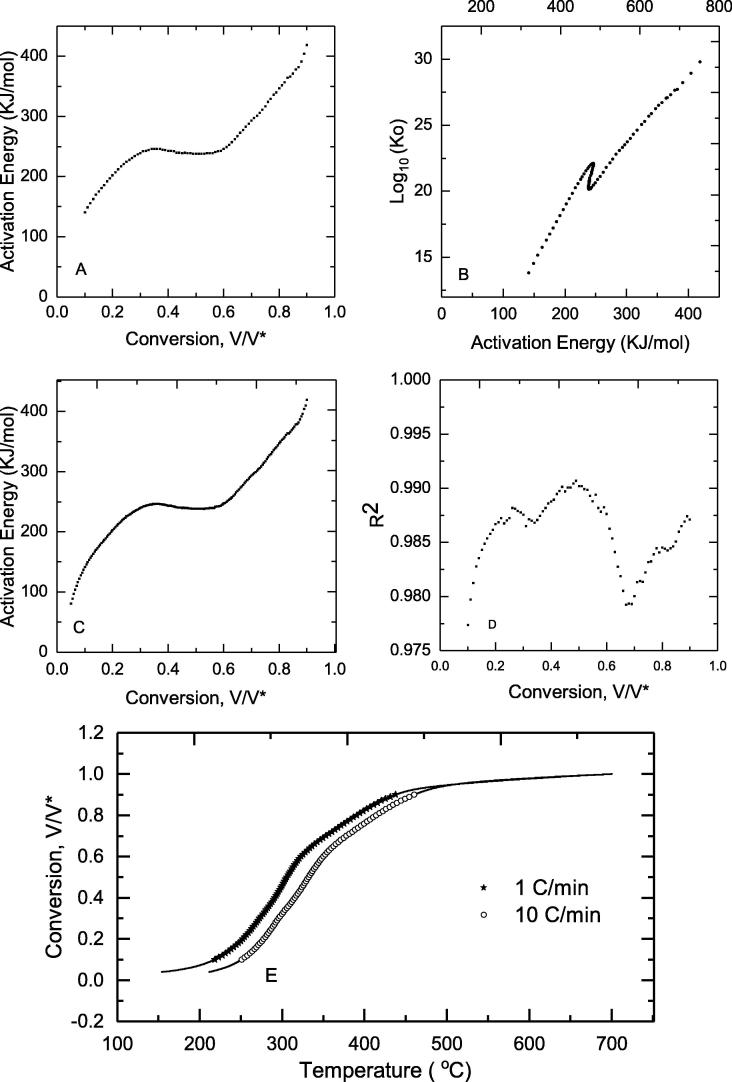
Pyrolysis kinetic modeling outputs. (A) Activation energy prediction from Miura-Maki DAEM model plotted against conversion (B) Logarithm of pre-exponential factor $k_{0}$ from Miura-Maki DAEM model (C) Activation energy prediction from isoconversional method (D) R^2^ value for linear Arrhenius plots used in Miura-Maki DAEM model, and (E) Predicted conversion values (symbols) plotted alongside experimental conversion data (solid lines) for 1 °C/min and 10 °C/min heating rates.

Linear plots of $\ln\left(\frac{\beta }{T^{2}}\right)vs.\left(\frac{1}{T}\right)$ resulted in high R^2^ values showing the validity of the approach with the data set used. Fig. 4D shows the R^2^ values for each of the selected conversion values. The activation energy determinations for both the DAEM and isoconversional methods produced results within a maximum of 0.8% of each other (Fig. 4(A) and (C)). The activation energy value ranged from 141 kJ/mol to 409 kJ/mol, for conversion values of 0.1–0.9. The Evalues initially increased from α of 0.1–0.3, then settled to a plateau, which was followed by an increase of E. The shape of the activation energy for the early and late conversion values could be indicative of elementary steps in the reaction not captured by the approach taken for analysis. The plateau value for both models (Isoconversional and DAEM) had an average of 241.5 kJ/mol ± 2.9 kJ/mol. The results of the Isoconversional and Miura-Maki methods are within a tenth of a kJ/mol of $E\left(\alpha \right)$ as shown by Fig. 4. Thus, it is assumed that the predicted values of $k_{0}\left(E\right)$ by Miura-Maki will be as accurate as those found from methods proposed by Vyazovkin and Lesnikovich (1988). The set of linear plots of $\ln\left(\frac{\beta }{T^{2}}\right)vs.\left(\frac{1}{T}\right)$ used to produce the kinetic parameters is included in the supplementary information. Though the Isoconversional method is a more robust method for estimating activation energy, the Miura-Maki method is less computationally intensive and simultaneously solves for E and $k_{0}\left(E\right)$; it is the preferable method for describing the kinetics of feces pyrolysis.

**Supplementary information:** 

The values of $k_{0}$ in min^−1^ were also obtained from Eq. (19). The values are shown in Fig. 4(B) and ranged from 10^15^ to 10^30^. The range of $k_{0}$ values determined for α of 0.3–0.6 were 10^21.0^ ± 10^0.7^ min^−1^ showing a narrow band corresponding to the flat portion of the activation energy (E) curve.

Validation of the Miura-Maki method in the determination of feces pyrolysis kinetics was undertaken by analyzing the accuracy of predictions by Eq. (13) using the solved values of $E,k_{0}\left(E\right),andf\left(E\right)$. Fig. 4(E) shows that the conversion values of the model prediction and the experimental data are very similar for both the 1 and 10 °C/min, further demonstrating the effectiveness of the Miura-Maki method for describing the kinetics of feces pyrolysis.

The activation energy values determined were higher than those reported by Othman et al. (2010) for raw sewage sludge, a study in which comparable experimental methods to the present study were used. Othman et al. found that activation energy varied from 150 to 200 kJ/mol for conversion ranges of 0.1–0.7. The difference is likely to be some of the degradation happening in the sewage as exhibited by the lower carbon content of 30% vs. this study’s 43%. Dry Fresh feces is known to be composed of about 17% plant like materials, 49% microorganisms, 5% sugars such as hexose, pentose, and cellulose, and a balance of other soluble components (Stephen and Cummings, 1980). Fat component of dry feces in normal individuals is expected to be 3 g per day as dry basis (Stephen and Cummings, 1980), and based on a 16% microbial fat content, about 75% of the fat is expected to be contained in the microbial component of feces. The first increasing portion of the activation energy curve ranges from 80 kJ/mol to 238 kJ/mol, with an average of 181 kJ/mol. This range is known to include Hemicellulose and Cellulose components (Vamvuka et al., 2003). Urban and Antal (1982) used a multi component method for undigested sludge pyrolysis kinetic data analysis and found average activation energy values of 130 and 250 kJ/mol for two components. The higher value from Urban and Antal’s study aligns well with our study and suggests that the higher activation energy component could be microorganisms.

One of the limitations of this study is that a few number of subjects were used for feces collection. It is known that the content of feces varies with diet, age, and health conditions. Dietary fiber has the most effect on the composition of feces through the action of increasing the activity of the microflora of the gut (Müller et al., 2018, Stephen and Cummings, 1980). The resulting effect is the increase in feces weight mainly due to increased microbial count in feces with an associated increase of nitrogen and fat content of feces. Serio and co-workers (Serio et al., 2002) present a study which looked at the pyrolytic evolution of gases from different animal manures including chicken, turkey, seabird, and cow. Their result indicated a coefficient of variation of 0.23–0.27 for the dry ash free mass yields of CO_2_, CH_4_, and CO at a 30 °C/min heating rate. In comparison, the coefficient of variation for the dry mass yield of CO_2_, CH_4_, and CO for the five heating rates used in the present work ranges from 0.19 to 0.32. It is thus expected that any variability of feces would likely not be as important as the variability expected from the heating rates utilized.

## 4 Conclusions

This study showed that non-condensable gases were released with an average yield of 20.9% on a mass basis for a slow pyrolysis of fresh dried feces. Methane and carbon dioxide dominated the gaseous release at mid-temperatures (∼450 °C) whereas hydrogen dominated at higher temperatures (above 600 °C). While slow pyrolysis is mainly intended for a solid char product, this study quantifies the energy from non-condensable gases produced during this process. Based on the estimated energy output, operation of a slow pyrolysis system at around 450 °C would be optimum. Gas utilization systems can be designed using the concentration of the pyrolysis gases reported in this study. Two methods for determining the kinetic parameters of feces pyrolysis were compared. It was found that the DAEM method proposed by Miura and Maki was able to predict the dependence of activation energy on conversion to within one percent of the more robust Isoconversional method. Conversions predicted by the Miura-Maki kinetic parameters based on many irreversible first order reactions for different heating rates were very close to the experimental values.
