# Supplementary material for: Pyrolysis of human feces: Gas yield analysis and kinetic modeling
Source: Waste Manag. 2018 Sep;79:214–22. doi: 10.1016/j.wasman.2018.07.020 (PMC6202436; doi:10.1016/j.wasman.2018.07.020)
Supplement: Supplementary file 1 [file mmc1.docx]

**Title:** Pyrolysis of human feces: gas yield analysis and kinetic modeling
**Journal:** Waste Management

Yacob Et Al. 2018

Supplementary Figures

Figure SI 1

Figure SI 1: Normalized ion current values of measured pyrolysis gases and rate of mass change DTG (1/min) plotted as a function of temperature for all five heating rates used.

Figure SI 2

Figure SI 2: Measured concentrations (ppm) of the pyrolysis gases plotted as a function of temperature for all the heating rates used.

Figure
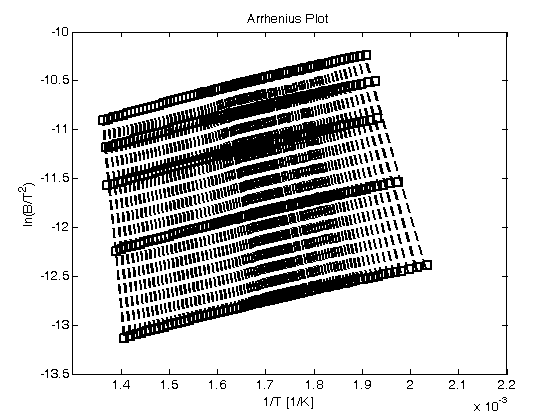
SI 3


Figure SI 3: Linear plots of $\ln\left( \frac{\beta}{T^{2}} \right)vs. \left( \frac{1}{T} \right)$ for selected conversion values
